# Assessment of the impact of heart failure on household economic well-being: a protocol

**DOI:** 10.12688/wellcomeopenres.16709.2

**Published:** 2021-11-09

**Authors:** Sivadasanpillai Harikrishnan, Sanjay Ganapathi, Salim Reethu, Ajay Bahl, Anand Katageri, Animesh Mishra, Anoop George Alex, Bhavesh Roy, Bishav Mohan, Hasit Joshi, Jabir Abdullakutty, Justin Paul, Maneesh Rai, Cholenahally Manjunath, Prakash C. Negi, Durgaprasad Rajasekhar, Rishi Sethi, Satyanarayan Routray, Radhakrishnan Shanmugasundaram, Sumanta Shekhar Padhi, Shyam Sunder Reddy P, Panniyammakal Jeemon

**Affiliations:** 1 ICMR Centre for Advanced Research and Excellence In Heart Failure (CARE-HF), Sree Chitra Tirunal Institute for Medical Sciences and Technology, Trivandrum, India; 2 Post Graduate Institute of Medical Education and Research, Chandigarh, India; 3 Sri Jayadeva Institute of Cardiovascular Sciences and Research, Kalaburagi, India; 4 North Eastern Indira Gandhi Regional Institute of Health and Medical Sciences, Shillong, India; 5 Christian Medical College Hospital, Vellore, India; 6 Zydus Hospital, Ahmedabad, India; 7 Dayanand Medical College Hospital, Ludhiana, India; 8 Apollo Hospitals International Ltd, Gandhinagar, India; 9 Lisie Hospital, Ernakulam, India; 10 Madras Medical College, Chennai, India; 11 KMC Hospital, Mangaluru, India; 12 Sri Jayadeva Institute of Cardiovascular Sciences and Research, Bengaluru, India; 13 Indira Gandhi Medical College Hospital, Shillong, India; 14 Sri Venkateswara Institute of Medical Sciences, Tirupati, India; 15 King George’s Medical University, Lucknow, India; 16 SCB Medical College, Cuttack, India; 17 PSG Institute of Medical Sciences and Research, Coimbatore, India; 18 MMI Narayana Multispeciality Hospital, Raipur, India; 19 Kims Hospitals Kondapur, Hyderabad, India

**Keywords:** Heart failure, catastrophic health expenditure, distress financing, out of the pocket expenditure

## Abstract

**Background:** Heart failure (HF), which is an emerging public health issue, adversely affects the strained health system in India. The adverse impact of HF on the economic well-being has been narrated in various anecdotal reports from India, with affected individuals and their dependents pushed into the vicious cycle of poverty. There is limited research quantifying how HF impacts the economic well-being of households from low- and middle-income countries.

**Methods:** We describe the methods of a detailed economic impact assessment of HF at the household level in India. The study will be initiated across 20 hospitals in India, which are part of the National heart Failure Registry (NHFR). The selected centres represent different regions in India, stratified based on the prevailing stages of epidemiological transition levels (ETLs). We will collect data from 1800 patients with acute decompensated HF and within 6-15 months follow-up from the time of initial admission. The data that we intend to collect will consist of a) household healthcare expenditure including out-of-pocket expenditure, b) financing mechanisms used by households and (c) the impoverishing effects of health expenditures including distress financing and catastrophic health expenditure. Trained staff at each centre will collect data by using a validated and structured interview schedule. The study will have 80% power to detect an 8% difference in the proportion of households experiencing catastrophic health expenditures between two ETL groups.  After considering a non-response rate of 5%, the target sample size is approximately 600 patients from each group and the total sample size is 1800 patients.

**Potential Impact:** Our study will provide information on catastrophic health spending, distress financing and household expenditure in heart failure patients. Our findings will help policy makers in understanding the micro-economic impact of HF in India and aid in allocation of appropriate resources for prevention and control of HF.

## Introduction

Globally, non-communicable diseases (NCDs) are one of the major causes of death and disability. Among NCDs, cardiovascular disease (CVD) is responsible for a major share of premature deaths and disability ^1^. Additionally, the burden of CVD is expected to grow in the coming years especially in the low- and middle-income countries (LMICs) ^2^. Since LMICs have the dual burden of disease – both from communicable and non-communicable diseases – NCDs exert a tremendous strain on the already challenged health systems ^3^.

Heart failure (HF) is a major contributor to the CVD burden, which consumes about 2% of the total health expenditure in many high-income countries ^4^. Available data suggest that HF is emerging as an important public health problem in LMICs. Given the resource intensive nature of HF management, the treatment is unaffordable to majority of the population in India, where the out-of-pocket spending for health ranges from 60–80% ^5, 6^. The effect of the growing burden of disease due to CVD including HF is not limited to the health sector but threatens the macro and micro economy as well. Additionally, HF occurs in India a decade earlier and in the most productive life years as compared to high-income countries ^7, 8^. Early mortality and disability in the productive life years due to HF is a loss to a nation’s productivity and negatively affects the economic conditions of households. Exceptionally low utilisation of device therapy noted in Indian patients with HF is likely related to non-affordability due to financial constraints ^9^. However, there is limited research quantifying how HF impacts the economic well-being of households in India ^9^.

We propose to conduct a study to assess the economic impact of HF at the individual and household level. The proposed study will specifically examine the impact of HF and related disabilities on household economic wellbeing." We will assess (a) household healthcare expenditure including out-of-pocket expenditure, (b) financing mechanisms used by households, (c) impoverishing effects of health expenditures, and (d) effect of HF on household income, productivity and functional limitations. We will examine each of the above items across socioeconomic and age groups, family size, and area of residence.

## Methods

### Ethical considerations

Our study is approved by the institutional ethics committee (IEC) of the Sree Chitra Tirunal Institute for Medical Sciences and Technology, Trivandrum (SCT/IEC/1313.6/DECEMBER-2018). Further, we will obtain approval from the IECs of individual participating centres before the commencement of the study. Written informed consent will be obtained from all participants.

### Study settings

The study will be developed under the platform of the National Heart Failure Registry (NHFR). The details of NHFR are published elsewhere ^10^. Briefly, 54 hospitals from different states of India are participating and recruiting patients in the NHFR. They register consecutive HF patients admitted in their hospitals during the study period of 3 years. Each participating centre is expected to register about 180 patients as part of NHFR.

We divided the NHFR sites into three groups based on the epidemiological transitional level (ETL) of their respective states. The ETL state groups are based on the ratio of disability adjusted life years (DALYs) from communicable, maternal, neonatal, and nutritional diseases to those from non-communicable diseases and injuries combined in 2016. The ETL is divided into 4 categories; the states with ratios of 0·56–0·75 (low ETLs), 0·41–0·55 (lower-middle ETLs), 0·31–0·40 (higher-middle ETLs), and less than 0·31 (high ETLs). For our study, we will group the low and lower-middle ETL (0·41–0·75) into one group, and retained the higher-middle ETL (0·31–0·40) and high ETL (<0·31) categories ^3, 10^. From each of the three selected regions based on the ETL classification, 6–8 hospitals will be identified. We will purposively select hospitals in both public and private domains (n=20) to give representation to both health sectors. The Sree Chitra Tirunal Institute for Medical Sciences and Technology, Trivandrum (SCTIMST), will act as the national coordinating centre for the study. The details of the participating centres are given in Table 1 in the extended data ^11^.

### Selection of study participants

The data will be collected from 90 consecutive patients enrolled into the NHFR from each of the selected 20 centres. Heart failure patients who have their routine clinical follow-up appointment 6–15 months after the date of index-admission will be eligible to take part in the study. The restriction of follow-up period after the acute admission date will enable us to collect the in-patient costs, procedure costs, and outpatient costs from all study participants. Further, it will also reduce the memory recall bias.

### Inclusion and exclusion criteria

We will use the European Society of Cardiology (ESC) 2016 guidelines for the diagnosis of HF ^12^.

The inclusion criteria for the study will be:

1) Patients aged between 18 and 70 years

2) Patients who had their first heart failure admission between 6 to 15 months before survey date

3) Resident of India

The following types of patients will be excluded from the study:

1) Patients who did not provide consent
2) Patients with concomitant illnesses such as:

• Malignancy
• Chronic systemic illness – haematological, collagen vascular and other immunological diseases, chronic infections on treatment / prophylaxis
• Primary renal disease on dialysis
• Post-cardiac transplantation patients
• Concurrent HIV, hepatotropic viral infections on treatment.

Patients who meet the above inclusion criteria will be identified in advance from the NHFR.

### Study tools

We adapted the existing tools used in a previous economic impact assessment study conducted in India ^13^ for other CVD outcomes. The domains covered under the questionnaire were as follows:

1. Demographic characteristics

2. Patient heart failure disease history and treatment expenditures

3. Assessment of functionality and productivity

4. Household characteristics

5. Household expenditure

6. Household assets 

***Validation of the questionnaire*.** The modified version of the questionnaire used by Huffman *et al.* ^13^ in LMIC was used by Daivadanam *et al*. ^5^ for a similar study on acute coronary syndromes in Trivandrum, Kerala. We modified the study tool used by Daivadanam *et al.* for assessing the micro-economic impact at the family level for HF patients in India. The modified questionnaire was subjected to a detailed validation process described below.

***Face validity*.** In order to ensure face validity, the questionnaire was given to a public health expert, practicing cardiologists (n=3), health policy expert (n=1) and heart failure patients of varying socio-economic class (n=5). Both regional languages and English versions were submitted to experts for ensuring translational validity. The questionnaire was subsequently piloted in 10 patients or their care givers and observed the response to each item. We removed four questions after the process of checking the face validity. Additionally, we added six questions based on expert’s opinion, modified and adapted eight questions and removed two questions that were not relevant to the current settings (the questions modified and adapted in the new questionnaire can be found in Table 2 in the extended data ^11^). Once the questionnaire in English and Malayalam were finalised, the English version was sent for translation to other Indian languages, depending upon the states, where the recruitment is planned.

***Translation to regional languages*.** The questionnaire was translated to nine different Indian languages. Translation was done by an agency who is well versed in the field ( Trans Lingua translational services, Bangalore, India). We aimed to achieve conceptual equivalence of a word or a phrase, and not a word-for-word translation. The meaning of sentences was adapted and translated to give the best meaning in a simple, clear and understandable manner. For achieving semantic equivalence, the translated versions were given to a group of language experts who were asked to rephrase every question in their own words or to narrate what they understood from each question. Additionally, each item in the regional language version was back translated to English by an independent person. The back translated English versions were checked for consistency. Conflicts were resolved by repeating this cycle one more time. Each item in the translated and back-translated questionnaires underwent strict verification and necessary corrections were done until both versions became agreeably consistent. The final version in each language will be used for data collection from the respective participating centres. The questionnaires can be found as extended data ^11^.

### Primary outcomes

Various indicators relating to the economic impact of HF on households will be estimated using data from the sample population. One important indicator is the proportion of households experiencing catastrophic health expenditures. Of primary interest is how the indicator of interest differs according to ETL group in a statistically significant way. It is important to ensure that there are adequate numbers of patients in each of the three ETL groups to be able to detect statistically significant differences between groups.

### Sample size calculation

Assuming a Type-1 error of 5%, power of 80%, 5% non-response rate, and 33% of the sample from each of the three ETL groups, 1800 patients (600 from each ETL groups) are required to detect an 8% difference in the primary outcome between any two ETL groups.

The expected rates of primary outcomes will be 50% and 60% for catastrophic health expenditure and distress financing, respectively ^5, 13^.

### Data collection

Participants will be interviewed when they come to the out-patient department for follow-up. Written informed consent will be taken from the patient, prior to collection of data. Trained staff will administer the structured interview schedule. The data collection will be done in printed paper form from participating centres (hospitals) and the data will be entered using electronic forms created in KoBoToolbox at the coordinating centre. The data will be protected for privacy with the following standard precautions. We will assign a unique identification number to all participating centres and for each enrolled patient, so that they will be further identified only by that number. Individual patient identification details will be collected and maintained only at the participating centres. For the ease of operation, a procedural manual was developed and circulated to each participating centres.

### Administration and management

All relevant documents will be sent to study centres for necessary ethics clearance and permissions. Designated staff will recruit patients, collect relevant data, by using the paper questionnaire. The collected data will be transmitted to the coordinating centre for further scrutiny. It will be verified for accuracy and completeness by the coordinator centre. Further, the coordinator centre will contact each participating centre weekly to get necessary updates of data collection and give necessary instructions to improve and maintain data quality. All the data entry queries will be answered and resolved by the coordinator centre. The Sree Chitra Tirunal Institute for Medical Sciences and Technology (SCTIMST) will act as the study coordinating centre.

### Data management and analysis

The data will be entered and analysed using statistical package SPSS version 17. Data analyses will be initiated only after data cleaning, quality checks and after identifying missing variables. We will generate a listing of data queries for the participating centres to resolve data-related issues monthly. The data will be saved in in-house server of SCTIMST. The authorised authority to view data will be the principal investigator and any other person as authorised by the principal investigator. A database lock will be employed to finalize the data set before the final statistical analyses. No statistical analyses will be conducted before the database lock, and no modification of data will be allowed after the database lock.

In order to describe the patient characteristics, the categorical variables will be presented as proportions. The distribution of the continuous variables will be checked, and normal distribution will be ensured before applying any parametric tests. Continuous variables will be presented as means with standard deviation (SD). If the continuous variables are not normally distributed, they will be presented as median with interquartile range (IQR). We will conduct descriptive analyses to explain the characteristics of the study population stratified by gender and location.

The key outcome variables will be compared across region, gender, income groups, types of hospitals etc. We will employ Chi Square test to compare proportions across study groups. Similarly, the difference in mean will be compared using independent t-test or analysis of variance (ANOVA) as appropriate. Multi-variate logistic regression models will be employed to understand the factors associated with outcome variable. The outcome variables are explained below.

**Figure 1.**
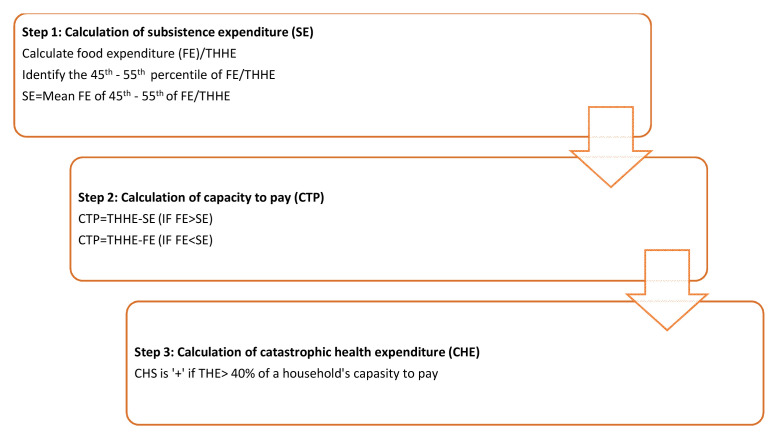
Steps in calculating catastrophic health expenditure. *FE-Food expenditure, THHE-Total household expenditure, SE-Subsistence expenditure, THE-Total health expenditure.

a) Total out-of-pocket (OPP) expenditure on health; includes all direct and some indirect health-related expenses incurred. Total OPP expenditures will include (a) direct costs of HF treatment: hospital fees, drugs, consultations, investigations, supplies, informal payment; and (b) indirect costs of HF treatment: travel, food, foregone wages, attendant costs. Reimbursements from third-party payers will be estimated and deducted.
b) Expenditures on HF treatment; includes direct and indirect expenditures related to HF diagnosis and treatment. Expenditure on HF treatment in the past 6 months will include (a) direct costs of HF treatment: hospital fees, drugs, consultations, investigations, supplies, informal payment; and (b) indirect costs of HF treatment: travel, food, forgone wages, attendant costs. Reimbursements from third-party payers will be estimated and deducted.
c) Distress financing: When faced with health shocks or high health expenditures, households engage in distress financing. This includes borrowing money from relatives/friends, selling assets (e.g. land) or taking loans. We will calculate the proportion of households within distress financing and gather their source of financing health care.
d) Catastrophic health expenditure: It will be estimated using the formula given below. A household’s health spending is considered catastrophic if annual out-of-pocket health expenditures comprise 40% or more of capacity to pay. Capacity to pay (CTP) refers to the non-subsistence expenditure which is the difference between the Total House-Hold Expenditure (THHE) of a household and their subsistence expenditure (SE). The catastrophic health expenditure calculation is described in detail in Figure 1.

Based on the data obtained from the questionnaire, individuals will be assigned to specific income/consumption groups. Households will be categorized into three socioeconomic status (SES) groups; low, middle and high at the time of analysis. Thus, each sampled patient will, according to their per capita household income, be placed in one of three income groups. The socioeconomic distribution of the indicators of interest across economic groups will be also examined.

## Discussion

There is limited data from LMICs on disease burden, and the economic burden of HF to households. We will generate national representative data of micro-economic burden due to HF from 1800 patients. The data generated from this project will be useful for policy makers and planners in allocation of resources and devising strategies to reduce out-of-pocket expenditure due to HF in India.

## Dissemination

After completing the data analysis, the team will publish key-findings in academic journals related to cardiology, health economics and outcome research. A project report will be presented to Indian Council of Medical Research (ICMR) for review and further dissemination through their web-portal and network. Policy briefs on study findings and their implications will be developed and circulated to policy makers at the state, regional and national level.

## Study status

The data collection for the study started in September 2019. The investigators are planning to complete the data collection by end of 2021.

## Data availability

### Underlying data

No data are associated with this article.

### Extended data

Figshare: Assessment of the Impact of Heart Failure on Household Economic Well-being: Background and Methods-study proforma. https://doi.org/10.6084/m9.figshare.14747976.v2

This project contains the following extended data:

- Economic impact questionnaire (3).docx (Questionnaire in English, list of participating centres, questions modified and adapted in the new questionnaire)
- CARE HF EC IM Tool_2.0-Gujarati.pdf
- CARE HF EC IM Tool_2.0-Hindi.pdf
- CARE HF ECONOMIC IMPACT Tool_2.0-Tamil.pdf
- CARE HF ECONOMIC IMPACT Tool_2.0-Kannada.pdf
- CARE HF ECONOMIC IMPACT Tool_2.0-Oriya.pdf
- CARE HF ECONOMIC IMPACT Tool_2.0-Telugu.pdf
- CARE_HF_ECONOMIC IMPACT Tool_2.0-Punjabi.pdf
- CARE-HF-ECONOMIC IMPACT_MALAYALAM (1).pdf

Data are available under the terms of the Creative Commons Attribution 4.0 International license (CC-BY 4.0).
