# Supplementary material for: Assessment of the impact of heart failure on household economic well-being: a protocol
Source: Wellcome Open Res. 2021 Nov 9;6:167. Originally published 2021 Jun 30. [Version 2] doi: 10.12688/wellcomeopenres.16709.2 (PMC8491152; doi:10.12688/wellcomeopenres.16709.2)
Supplement: Peer review history

# Peer review: Reviewer response for version 1

**DOI:** 10.21956/wellcomeopenres.18426.r44702

**Published:** 2021-10-04

**Authors:** Santhosh Koshy

**Affiliations:** 1 Division of Cardiovascular Medicine, Texas Tech University Health Sciences Center, Lubbock, Texas, USA

The article just describes the hypothesis and methodology of a study that is being planned to be conducted. The article does not include analysis of any data and hence should be considered primarily as a descriptive manuscript. It would have been better if the hypothesis and methodology were published along with the data analysis and conclusion. 

 The hypothesis of the study is not well understood. It is understood that the comparison is made between two ETL groups as described in the method section. Is the hypothesis that the higher ETL levels have less economic impact with the disease? The actual hypothesis that is being tested should be clearly mentioned in the manuscript. 

 The impact section of the manuscript is misleading and sounds as if there was a conclusion already made that would help policy makers to create policies. I would recommend rewriting the last paragraph in Abstract as 'conclusion' (rather than titling it is as impact) and mention that the intent for this publication is to detail the hypothesis and methodology of a large-scale multi-center high impact study that is planned. 

 Since this is an open-label study, authors have to detail the potential bias that can occur and what are the steps taken to mitigate the impact of bias. There may be an intent to amplify the actual economic impact. 

 Other suggestions:

- The following sentence in the Introduction is redundant. "For example, one-third of CVD deaths occur in the working and child-rearing age populations ^2^."
- I would recommend rewording the sentences in the introduction as "Exceptionally low utilisation of device therapy is noted in Indian patients with HF is likely related to non-affordability due to financial constraints ^9^." Then delete the sentence "One of the reasons for low uptake of guideline-directed device therapy could be non-affordability of the treatment options."
- I would recommend rewording the sentence in the introduction to "The proposed study will specifically examine the impact of HF and related disabilities on household economic well-being." 
- I would recommend reviewing the manuscript again and condense/concise the writing as much as possible to make it easy for the reader. 

Is the study design appropriate for the research question?

Yes

Is the rationale for, and objectives of, the study clearly described?

Partly

Are sufficient details of the methods provided to allow replication by others?

Yes

Are the datasets clearly presented in a useable and accessible format?

Not applicable

Reviewer Expertise:

Intravascular imaging, heart failure, Interventional cardiology

I confirm that I have read this submission and believe that I have an appropriate level of expertise to confirm that it is of an acceptable scientific standard.

# Response

**Published:** 2021-11-01

**Authors:** Panniyammakal Jeemon

**Affiliations:** Sree Chitra Tirunal Institute for Medical Sciences and Technology, Trivandrum, India

Thank you very much for approving our manuscript and sending the detailed comments. Please find below our detailed point-by point response to the comments. 

 Comment: **The article just describes the hypothesis and methodology of a study that is being planned to be conducted. The article does not include analysis of any data and hence should be considered primarily as a descriptive manuscript. It would have been better if the hypothesis and methodology were published along with the data analysis and conclusion.**

**Response: **This is a protocol paper and we will publish the results of data analysis after the completion of the study. This has been clearly stated in the title of the study. 

**Comment: The hypothesis of the study is not well understood. It is understood that the comparison is made between two ETL groups as described in the method section. Is the hypothesis that the higher ETL levels have less economic impact with the disease? The actual hypothesis that is being tested should be clearly mentioned in the manuscript.**

**Response: **We divided the study sites into three groups based on the epidemiological transitional level (ETL) of their respective states. Our primary interest is to identity how the indicators of economic wellbeing differs according to ETL groups.

**Comment: The impact section of the manuscript is misleading and sounds as if there was a conclusion already made that would help policy makers to create policies. I would recommend rewriting the last paragraph in Abstract as 'conclusion' (rather than titling it is as impact) and mention that the intent for this publication is to detail the hypothesis and methodology of a large-scale multi-center high impact study that is planned.**

 Response: To avoid confusion we changed the "impact" section to "Potential impact". In a protocol paper we do not normally provide a "Conclusion" section. We have also modified the paragraph to avoid any confusion. The revised paragraph is as follows: 

 "Our study will provide information on catastrophic health spending, distress financing and household expenditure in heart failure patients. Our findings will help policy makers in understanding the micro-economic impact of HF in India and aid in allocation of appropriate resources for prevention and control of HF". 

**Comment: Since this is an open-label study, authors have to detail the potential bias that can occur and what are the steps taken to mitigate the impact of bias. There may be an intent to amplify the actual economic impact.**

 Response: As this is a cross sectional study it will have all the possible biases encountered in a cross-sectional study. The patients for this study will be recruited from the hospital registered in the national heart failure registry. Therefore, patients visiting other hospitals and patients in the community who may not seek care due to various reasons will be excluded from this study.

**Comment: The following sentence in the Introduction is redundant. "For example, one-third of CVD deaths occur in the working and child-rearing age populations."**

 Response: This sentence has been removed from the manuscript.

**Comment: I would recommend rewording the sentences in the introduction as "Exceptionally low utilisation of device therapy is noted in Indian patients with HF is likely related to non-affordability due to financial constraints." Then delete the sentence "One of the reasons for low uptake of guideline-directed device therapy could be non-affordability of the treatment** **options."** 

 Response: We have modified the statement as suggested by the reviewer. 

**Comment: I would recommend rewording the sentence in the introduction to "The proposed study will specifically examine the impact of HF and related disabilities on household economic wellbeing."**

 Response: We have modified the statement as suggested by the reviewer.

# Peer review: Reviewer response for version 1

**DOI:** 10.21956/wellcomeopenres.18426.r45363

**Published:** 2021-08-23

**Authors:** Hisham Moosan

**Affiliations:** 1 Indian Council of Medical Research, New Delhi, Delhi, India

A few suggestions for the authors;

**In the Abstract:**

- It may be good to mention the usage of the NFHR platform, as mentioned in the study settings.

**In the Sample size section:**

- All are expressed in percentages, except for power, which is shown as a proportion of 1. A uniform usage would seem more appropriate.

**In the Data collection section:**

- "the data will be entered in KoBo Toolbox" seems a little incorrect. Is it that the data will be entered into electronic forms, created using KoboToolBox? 

**In the Administration and management section:**

- It is said that - *"Designated staff will recruit patients, **collect **relevant data, **by using either a paper questionnaire or the online platform**".* Whereas in the earlier Data collection section it is mentioned *"The data collection **will be done in printed paper form from participating centres** (hospitals) and the data will be entered in KoBoToolbox in the coordinating centre in order to avoid errors in data entry."* Though it is slightly understandable that the former quote may have been referring to both the data collection and entry process, it may be best to reword it slightly to avoid confusion.

Is the study design appropriate for the research question?

Yes

Is the rationale for, and objectives of, the study clearly described?

Yes

Are sufficient details of the methods provided to allow replication by others?

Yes

Are the datasets clearly presented in a useable and accessible format?

Not applicable

Reviewer Expertise:

Epidemiology, Health Economics, Qualitative Research, health Policy and Systems Research

I confirm that I have read this submission and believe that I have an appropriate level of expertise to confirm that it is of an acceptable scientific standard.

# Response

**Published:** 2021-11-01

**Authors:** Panniyammakal Jeemon

**Affiliations:** Sree Chitra Tirunal Institute for Medical Sciences and Technology, Trivandrum, India

Thank you very much for approving our manuscript. We have added in the abstract that the study sites are part of the National Heart Failure Registry. We have also changed power in the sample size statement from 0.8 to 80%. The comment on the data entry using **KoboToolBox **is accepted and we have made appropriate changes in the manuscript text.
